# Supplementary material for: The implementation of a rapid sample preparation method for the detection of SARS-CoV-2 in a diagnostic laboratory in South Africa
Source: PLoS One. 2020 Oct 20;15(10):e0241029. doi: 10.1371/journal.pone.0241029 (PMC7575110; doi:10.1371/journal.pone.0241029)
Supplement: Attachment — Submitted filename: Response to Reviewers .docx [file pone.0241029.s004.docx]

# Response to Reviewers

Manuscript: PONE-D-20-24160R1

The implementation of a rapid sample preparation method for the detection of SARS-CoV-2 in a diagnostic laboratory in South Africa

Thank you for the review of our manuscript. We have prepared responses to the comments provided in addition to a revised manuscript.

## Editor’s comments

1. ***Table 2 is redundant since here exactly the same mixture scheme is used as in Table 1, with the difference that only 2 μl input in stead of 3 μl was used (which is compensated for by the water volume). I therefor advise to remove Table 2 and to add in the text after 'with a decreased sample volume' '2 μl in stead of 3 μl' (page 7, line 134).***

Response:

The manuscript has been appropriately updated.

***2. Please replace 'greater' by 'higher' before 'mean Ct value'***

Response:

The manuscript has been appropriately updated.
